# Introducing a brain-computer interface to facilitate intraoperative medical imaging control – a feasibility study

**DOI:** 10.1186/s12891-022-05384-9

**Published:** 2022-07-22

**Authors:** Hooman Esfandiari, Pascal Troxler, Sandro Hodel, Daniel Suter, Mazda Farshad, Collaboration Group, Nicola Cavalcanti, Oliver Wetzel, Sylvano Mania, Frederic Cornaz, Farah Selman, Method Kabelitz, Christoph Zindel, Sabrina Weber, Samuel Haupt, Philipp Fürnstahl

**Affiliations:** 1 Research in Orthopedic Computer Science (ROCS), Balgrist University Hospital, University of Zurich, Zurich, Switzerland; 2 Department of Orthopaedic Surgery, Balgrist University Hospital, University of Zurich, Zurich, Switzerland

**Keywords:** Brain computer interface, Medical image, Surgery, Image control, Display, Image manipulation

## Abstract

**Background:**

Safe and accurate execution of surgeries to date mainly rely on preoperative plans generated based on preoperative imaging. Frequent intraoperative interaction with such patient images during the intervention is needed, which is currently a cumbersome process given that such images are generally displayed on peripheral two-dimensional (2D) monitors and controlled through interface devices that are outside the sterile filed. This study proposes a new medical image control concept based on a Brain Computer Interface (BCI) that allows for hands-free and direct image manipulation without relying on gesture recognition methods or voice commands.

**Method:**

A software environment was designed for displaying three-dimensional (3D) patient images onto external monitors, with the functionality of hands-free image manipulation based on the user’s brain signals detected by the BCI device (i.e., visually evoked signals). In a user study, ten orthopedic surgeons completed a series of standardized image manipulation tasks to navigate and locate predefined 3D points in a Computer Tomography (CT) image using the developed interface. Accuracy was assessed as the mean error between the predefined locations (ground truth) and the navigated locations by the surgeons. All surgeons rated the performance and potential intraoperative usability in a standardized survey using a five-point Likert scale (1 = strongly disagree to 5 = strongly agree).

**Results:**

When using the developed interface, the mean image control error was 15.51 mm (SD: 9.57). The user's acceptance was rated with a Likert score of 4.07 (SD: 0.96) while the overall impressions of the interface was rated as 3.77 (SD: 1.02) by the users. We observed a significant correlation between the users' overall impression and the calibration score they achieved.

**Conclusions:**

The use of the developed BCI, that allowed for a purely brain-guided medical image control, yielded promising results, and showed its potential for future intraoperative applications. The major limitation to overcome was noted as the interaction delay.

**Supplementary Information:**

The online version contains supplementary material available at 10.1186/s12891-022-05384-9.

## Background

**Fig. 1:**
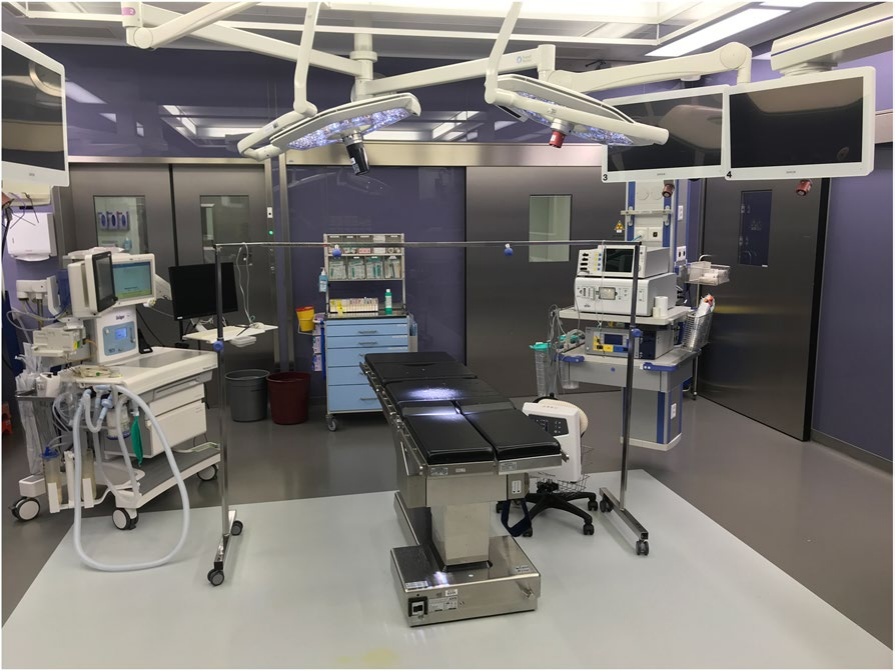
A typical operating room at the Balgrist University Hospital. Peripheral monitors can be seen outside the operating area

Surgical planning, navigation and execution is heavily dependent on medical image modalities, including radiography, Computer Tomography (CT) and Magnetic Resonance Imaging (MRI) [1]. Such images are generally stored through the Picture Archiving and Communication System (PACS) and presented to the operating team via 2D monitors. The modern Operating Room (OR) represents a challenging environment for interaction with imaging modalities (Fig. 1) and as stated in [2, 3], inadequate data presentation can be noted as a major workflow bottleneck inside the OR. This can be attributed to multiple factors such as missing spatial context when viewing medical images on 2D monitors [4] and the use of non-sterile input devices for image control such as, keyboard, mouse and touch screens. These conventional input devices can even be a reservoir for pathogens [5]. Scrubbed surgeons are generally not able to touch such input devices and are often forced to request another member of the operating team to act as a proxy and interact with the medical images [6]. This often results in delay and frustration as precise 3D manipulation of medical images while solely relying on verbal commands is a cumbersome undertaking [7].

Given the ever-increasing presence of advanced medical technologies inside the operating room, there is currently a high demand for intuitive and touchless human–computer interfaces that allow for seamless interaction with such devices, while maintaining the integrity of the sterile filed [8].

### Related Work

To overcome the abovementioned limitations, different touch-less interaction methods have been proposed that allow for direct image manipulation inside an operating room using gesture- or speech recognition technologies. One of the earliest examples of the vision-based gesture recognition technologies was presented in [9], where the authors developed a non-contact mouse for intraoperative use by detecting the surgeon's gestures based on a stereo camera setup. This was followed by several other publications that utilized image-based gesture recognition for medical image manipulation [10]. More recently, conceptually similar approaches haven been introduced that provide the possibility of remote, touch-less interaction with medical imagery based on gesture recognition using depth (i.e., RGB-D) sensors (e.g., [11–16]). Performing such gestures requires certain movements of either one or both hands, rendering such technologies limited for interventions where both of the surgeons' hands are occupied. Furthermore, such methods generally rely on outside-in tracking of the surgeon's gestures by placing a sensor next to each image modality of interest. This can result into an even more cluttered operating theater as multiple sensors are needed to interact with each image modality. Additionally, the required user gestures can be perceived as non-intuitive given that the user (i.e., the surgeon) should learn them for a smooth interaction experience with the technology [17]. While still relying on user's physical gestures, the authors in [18] proposed an inside-out gesture recognition method for medical image manipulation based a wearable RGB-D sensor. This alleviated the need for multiple gesture recognition sensors, by relying on a single head-mounted depth sensor. However, the system required user-specific and display-specific calibration steps and placement of external recognizable patterns (i.e., QR codes) on each display monitor. Further studies introduced a commercially available hand-tracking product (Leap Motion; San Francisco, United States) [19, 20] for the purpose of touch-less medical image control. As an overarching limitation associated to the abovementioned methods, gesture recognition based on external sensors can suffer from line-of-sight issues specially in the OR's crowded environment.

A parallel line of technology was developed in [21] where inertial measurement sensors were used to identify the user's gesture. Although these techniques have a smaller physical footprint in the OR and do not suffer from line-of-sight issues, they generally require a training phase based on a pre-acquired set of data and are attributed to the same limitations of gesture intuitiveness.

Speech recognition methods for medical image manipulation were presented in [13]; however, there can be substantial concerns with the efficiency of such algorithms in a noisy environment of an operating theater. In fact, the noise pollution inside the OR has been reported to be higher than the safe noise thresholds defined by the World Health Organization (WHO) [22]. Additionally, and in our own experience, relying on voice commands in the operating room environment can be a challenging undertaking even when input microphones are not covered.

### Contributions

Brain-Computer Interface (BCI) has been an active filed of research in the past decades with the promise of providing non-muscular means for communication of the users and machines [23]. Recent advances in signal processing and artificial intelligence have resulted in adoption of BCI systems in a variety of applications [24–28]. As a particular use-case of BCI in healthcare, researchers have extensively investigated the feasibility of BCI interfaces for rehabilitation medicine [29, 30]. However, the form-factor of the developed hardware and the specialized design of their associated interfaces have made it difficult to translate such technologies for intraoperative applications. However, with the recent introduction of consumer-grade BCI devices, we believe that the emerging BCI sensor technology is a suitable choice for the specific use case of intraoperative medical image manipulation, given that they do not rely on recognizing the surgeons' demands through external means of communication (e.g., hand movements, voice commands or foot pedals), but rather, detect the surgeon's desire directly by measuring their brain activity. Using the direct communication channel provided by BCI technology, the abovementioned shortcomings of the state-of-the-art techniques for touch-less image manipulation can be addressed. In this study, we present what we believe to be the first adoption of human-brain interface technology for intraoperative medical image manipulation. We developed a software environment that could provide touch-less and hands-free medical image control through real-time communication with a consumer-grade BCI device worn by the surgeon. The usability of our technique was assessed by orthopedic surgeons at our institution in a systematic fashion and metrics such as response time, usability, comfort, and accuracy were evaluated.

## Methods

### Choice of Sensor

**Fig. 2:**
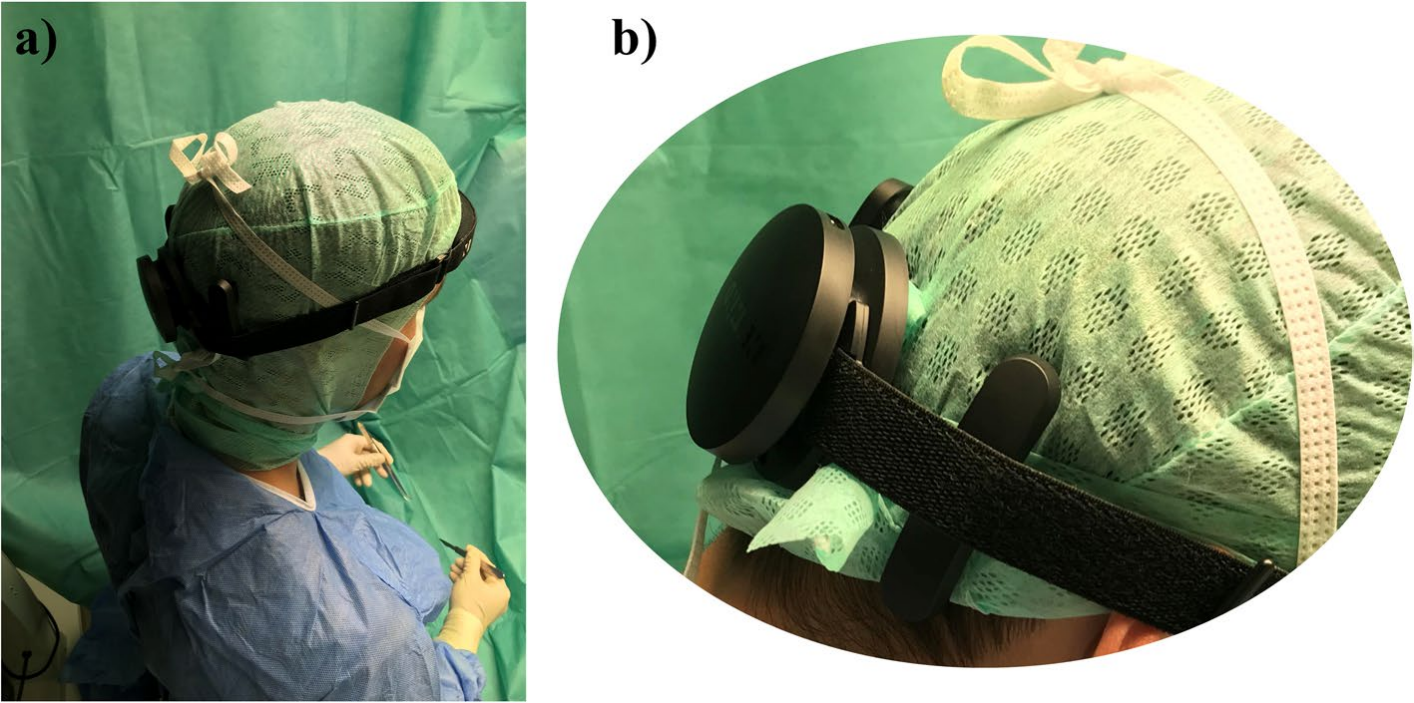
a) The BCI sensor being worn in a surgical setting by a surgeon. b) the position of the sensor on the surgeon's scull. Note that the sensor is worn over the surgical cap for visualization purposes but for the intended use-case, the sensor must be worn under the cap

Visually Evoked Potentials (VEPs) are brain activity modulations that take place in the visual cortex after being exposed to a visual stimulus [31], which can be robustly detected [32]. Building up on this technology, a new consumer-grade product was released recently that is capable of monitoring the brain activity using a small form-factor wearable sensor technology (NextMind; Paris, France, [33]). This device utilizes small dry electrodes that are in contact with the user's scull to monitor electrical activity in the visual cortex based on the Steady-State Visually Evoked Potentials (SSVEP) concept. This sensor technology is non-invasive and lightweight making it comfortable to be worn under a surgical cap (Fig. 2). To use this device as a computer interface, special buttons with a unique flickering visual patterns have to be implemented into the user interface, which can send corresponding software signals once the user wearing the sensor looks at them with appropriate level of contextual attention. This device is shipped with a Software Development Kit (SDK), sending appropriate software signals to be used in development of custom applications. Given that this device meets the clinical and application-specific requirements of our target application to a great extent, we used its hardware + SDK platform to develop the medical image manipulation application.

### Software Application Design

An application for intraoperative control of radiological images was designed using the abovementioned BCI platform and incorporating the functionality of the state-of-the-art PACS viewers. Given that the surgeons are familiar with the common PACS viewers to visualize the medical images in separate windows along different slice direction (coronal, sagittal and axial) and scroll through the slices with standard computer interfaces (e.g., computer mouse and keyboard), we considered them as the baseline for the design of our software application and developed a BCI-controllable medical image viewer software that allowed for touchless and gesture-free image manipulation while offering similar functionalities as a PACS image viewer software. After conducting an interview with a lead spine surgeon, we established the basic outline of the software application and the corresponding interface to best suit the communicated clinical needs. This software was developed by the authors and independent to the manufacturer of the BCI hardware. Details regarding the programming environment and the utilized libraries are included in Programming Environment section. This software was later used in the conducted user study (section User Study). The primary specifications of this software application were defined through direct consultations with our clinical collaborators and were iteratively refined through procurement of their feedback during several demo sessions. In each demo session, our clinical collaborators used the developed interface to navigate within the 3D medical images and to land on their desired anatomical markers. This was followed by one-on-one interview at the end of each demo session, during which our collaborating clinicians identified essential modifications to the software application for use in surgical conditions. Although the interface can be used for any 2D and 3D imaging modality, the herein specifications are explained for the use case of displaying patient CT scans used during spine surgery.

**Fig. 3:**
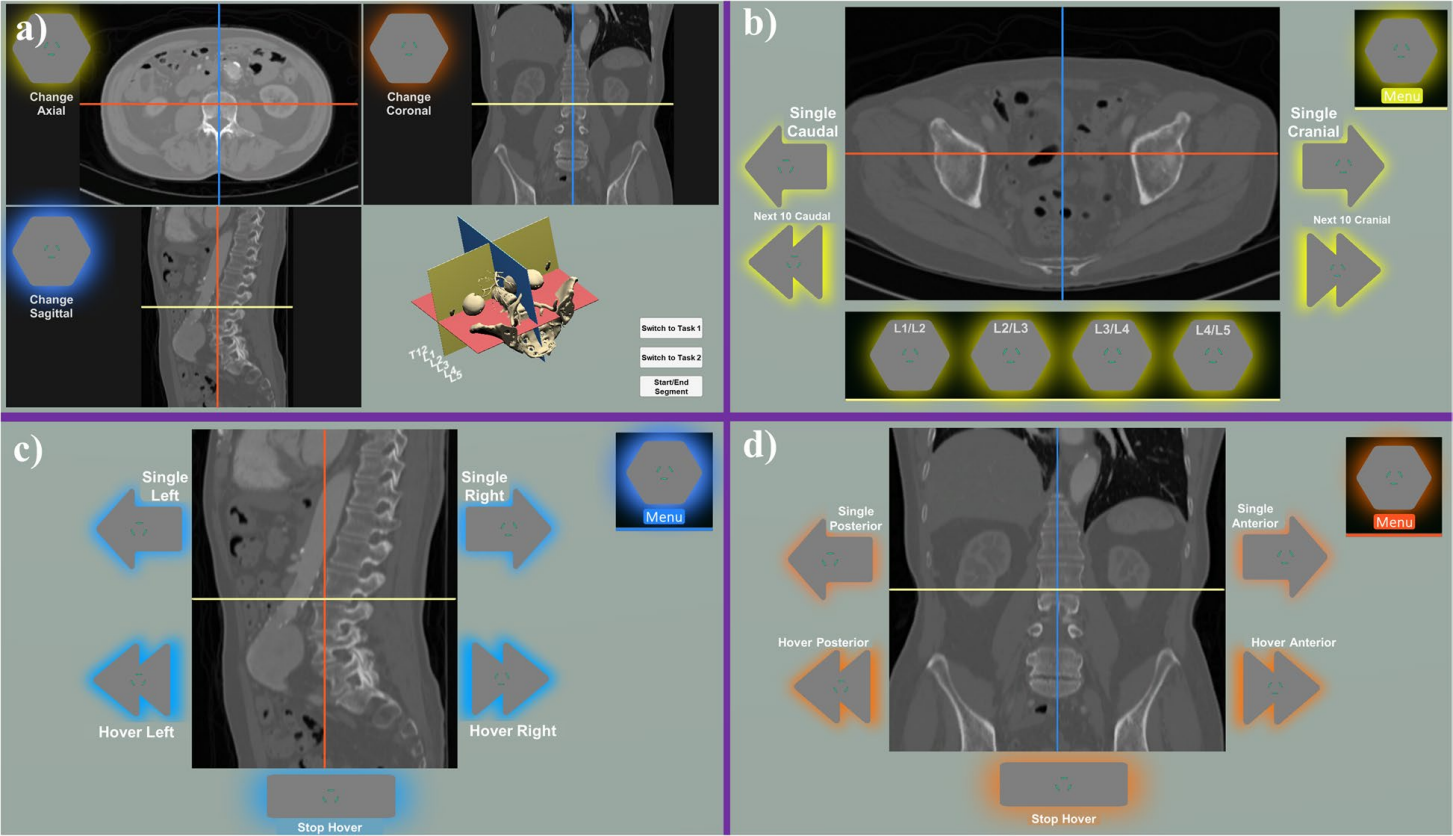
Image control interface. a) the global view displaying the current position of the axial, coronal and sagittal views. b) the axial view including the pre-defined landmark controls as well as the next 10 slice control. c) the sagittal view including the single slice scroll and hover scroll functionality. d) the coronal view including the single slice scroll and hover scroll functionality

Similar to standard PACS viewers, a global view (i.e., main menu; Fig. 3-a) contained the three windows for displaying slices along each anatomical direction (coronal, sagittal and axial) as well as a 3D display showing the volume of the CT scan. The interface displayed the application on two monitors (primary and secondary) and by looking at each of the corresponding buttons in the global view, the user could switch the primary view to the desired axis. Once the primary display was setup; the secondary display showed the two other slice directions (e.g., primary: axial – secondary: coronal and sagittal). Once on a given slice, the user could navigate from the current slice to the immediately adjacent slices by looking at the "single arrow" buttons. In order to find the most recent viewing positing in the CT scan, Crosshairs were implemented to indicate the slice position of one slice view in the two others. Furthermore, each slice view along with its corresponding crosshair was assigned a unique color seen as a colored halo effect around the buttons of that slice view (axial: yellow, coronal: orange and sagittal: blue). Inside the axial slice view, the user could navigate to dominant anatomical landmarks that were predefined in the patient CT scan. This was designed to facilitate the navigation into most important anatomical areas (as defined by our consulting surgeons). For the presented use case of spine surgery, these landmarks were defined as the intervertebral disc space in the lumbar region (Fig. 3-b). Hover buttons were introduced on the coronal and the sagittal slice views that initiated a "free-move" along a given direction, which could be stopped by the user by looking at the "stop hover" button (Fig. 3-c,d). This was designed in a way that successive activations of the hover button would result in increase of the "free-move" speed. Based on the feedback from our clinical collaborators, the controls in the axial slice view should be more involved; therefore, we exchanged the hover functionality in this view to navigation in the cranial-caudal axis by a definite number of slices (Fig. 3-b).

### User Study

The primary objective of this prospective user study was to evaluate the feasibility of the developed imaging control interface in a simulated surgery setting. For this, we recruited ten orthopedic resident surgeons at Balgrist University Hospital from August 2021 to December 2021. Eight male and two female surgeons with a mean age of 33 years (range 28 to 36) who had completed a mean of 51 months (range 23 to 80) of their orthopedic residency program. Each participant completed a series of image control tasks using the developed interface and the software application recorded each individual's performance.

As per the manufacturer's recommendation, each participant underwent an initial calibration process of the BCI device. This one-time calibration process was performed for each participant at the beginning of their session and a calibration score (range 1: poor – 5: excellent) was calculated for each participant. Each participant was given three attempts to reach a minimum calibration score of 3 and additional two attempts to reach a minimum score of 2. After the calibration phase, the participants were allowed to familiarize themselves with the hardware and software interface and perform provisional image controls for 10 min.

**Fig. 4:**
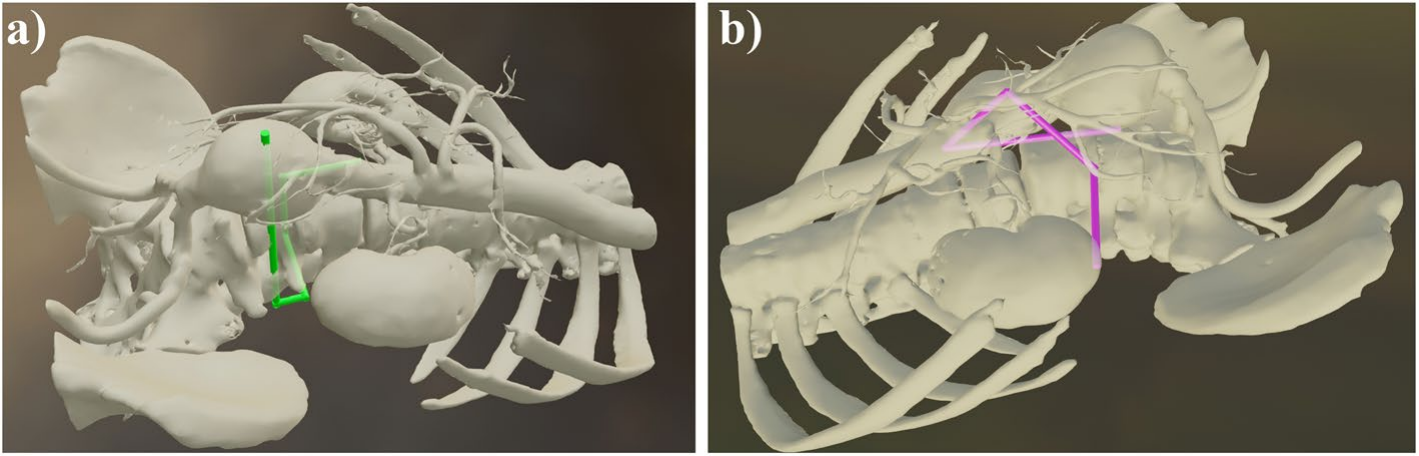
a) an example of a simple trajectory (task 1); b) an example of a difficult trajectory (task2)

As their primary task, the participants were asked to navigate to a predefined anatomical location in the patient CT scan while trying to follow a specific pre-defined trajectory to the best of their ability. This trajectory consisted of individual segments and had two different levels of difficulty. During the first task, the trajectory was constrained into segments that were strictly orthogonal to one of the slice directions (i.e., requiring the participant to only move in one of the axials, coronal or sagittal directions; Fig. 4-a). This constraint was lifted for the second and more difficult task, where the trajectories were designed in a way that a combination of movements along the three primary axes was needed for following each segment (Fig. 4-b).

**Fig. 5:**
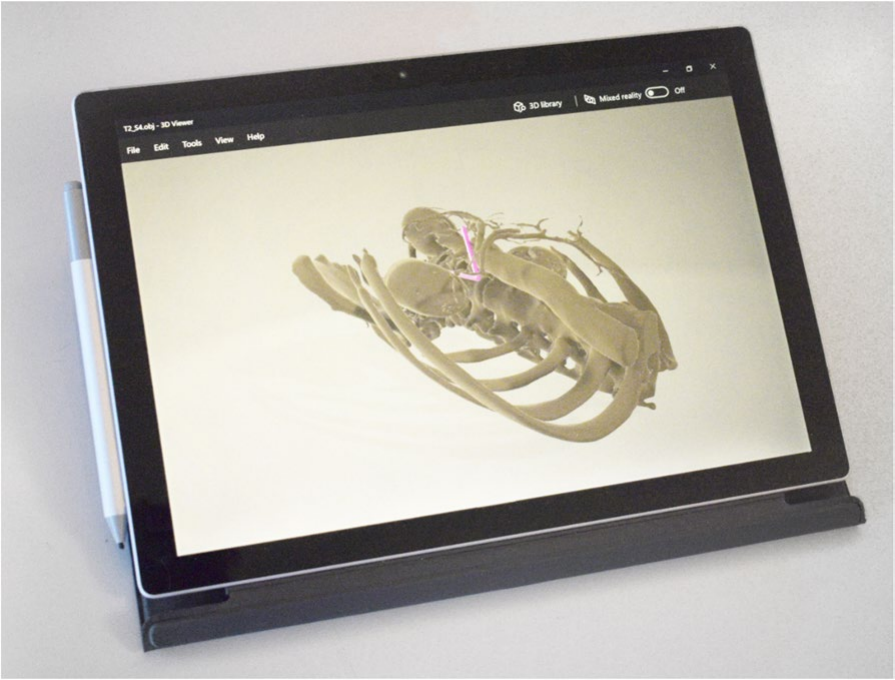
Trajectory visualization on a touchscreen tablet

Before starting the tasks, the participants were presented with the 3D representation of the desired trajectory overlaid on the patient's 3D model and displayed on a touchscreen tablet (Fig. 5). For better comprehension, the participant could see the desired trajectory displayed on the tablet from different view angles by rotating the scene, panning, and zooming in–out. After this inspection phase, the participants were blinded to the desired trajectory and were asked to follow it using only the developed interface.

While trying to follow each trajectory segment, the participant's trajectory and time was recorded in the background for the retrospective processing of their performance. Once satisfied with the end-point of their trajectory segment, the participants could choose to move on to the next segment. In the post-processing phase, and for each trajectory segment, we calculated the Euclidian distance between the participant's confirmed end-point and the corresponding pre-defined end-point (i.e., image control error).

After completion of the image control tasks, we asked the participants to evaluate the performance and the feasibility of the developed interface with respect to the following criteria: acceptance, input/output task, software application and overall personal impression (Additional file 1: User study questionnaire). This questionnaire consisted of 19 questions and the responses were recorded on a 5-point Likert scale. Responses were scored as strongly agree (5), agree (4), neutral (3), disagree (2) and strongly disagree (1).

Data related to descriptive statistics are presented as mean, Standard Deviation (SD) and range. In order to determine if there is a significant difference in the participants' image control errors between the two tasks, after checking for the normality of the data through Kolmogorov–Smirnov and Shapiro–Wilk tests, we ran a Wilcoxon's signed-rank test (significance was set at < 0.05). Correlation between rating scores and calibration score were analyzed using Spearman's rank correlation (*r*_*s*_) (significance was set at < 0.05).

### Programming Environment

The software was developed using the Unity engine editor (2019.4.20f1). The NextMind SDK was used that provided a high-level Application Programming Interface (API) to create the input and callback events. The data post-processing was done using Python 3.8 and the Matplotlib library was used for data visualization. Statistical analyses were performed using Microsoft Excel 2019. The image control tasks were performed on an anonymized, publicly available human CT scan (the sample dataset available in 3D Slicer software)^1^ with an in-plane resolution of 0.742 mm × 0.742 mm and slice thickness of 1.5 mm.

## Results

Mean calibration score of the BCI device was 3.7 (STD:0.8, range 3.0–5.0). In Table 1, the individual participants' calibration score and image control error (mm) is reported for both of the image control tasks. On average, the navigation error for task one (easier task) across all the participants was 16.9 mm (SD: 9.7) while the same error for task two (harder task) was 13.4 mm (SD: 9.0).

**Table 1:** Quantitative results of the participants' performance in the image control tasks

	**Participant**	**P1**	**P2**	**P3**	**P4**	**P5**	**P6**	**P7**	**P8**	**P9**	**P10**
	**Calibration score**	4	5	3	4	3	3	3	3	4	5
Task 1	**Mean** **Error (mm)** **(SD)**	10.1 (2.5)	8.0 (2.3)	7.2 (8.5)	12.2 (3.0)	19.6 (12.6)	16.7 (7.8)	14.8 (6.5)	17.0 (9.5)	16.3 (5.5)	23.3 (8.9)
	**Time (s)**	90.8	102.2	64.0	27.6	87.2	78.4	70.8	27.8	34.8	85.8
Task 2	**Mean** **Error (mm)** **(SD)**	11.8 (2.6)	8.3 (2.7)	17.1 (5.6)	7.6 (2.6)	16.9 (9.0)	10.2 (3.2)	22.2 (16.7)	22.2 (7.4)	7.4 (2.8)	21.8 (6.0)
	**Time (s)**	193.2	169.0	101.5	174.0	267.5	191.5	94.7	72.0	100.2	68.7

**Fig. 6:**
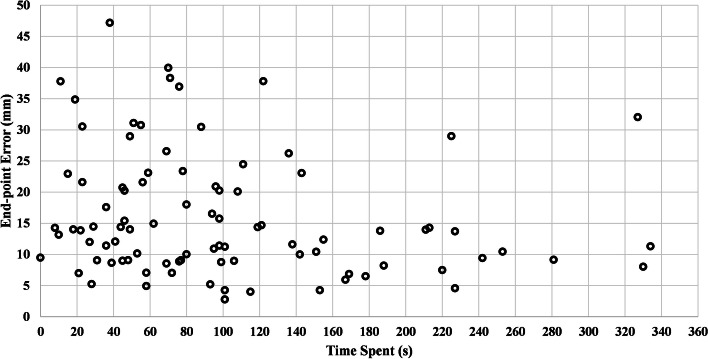
Image control error for navigating to each end-point (8 end points × 10 participants) versus the time required for completion of image control

By comparing all the end-point errors for task one (10 participants × 5 end-points) and task two (10 participants × 4 end-points) through a Wilcoxon's signed-ranks test (the data was determined to not follow a normal distribution by both the Kolmogrov-Smirnov and the Shapiro–Wilk tests), we concluded that there is not a statistically significant difference in participants' performance between the two tasks (*p* = 0.07). The errors for navigating to specific end-points and the corresponding time that the participants took to reach to that end-point is illustrated in Fig. 6.

**Fig. 7:**
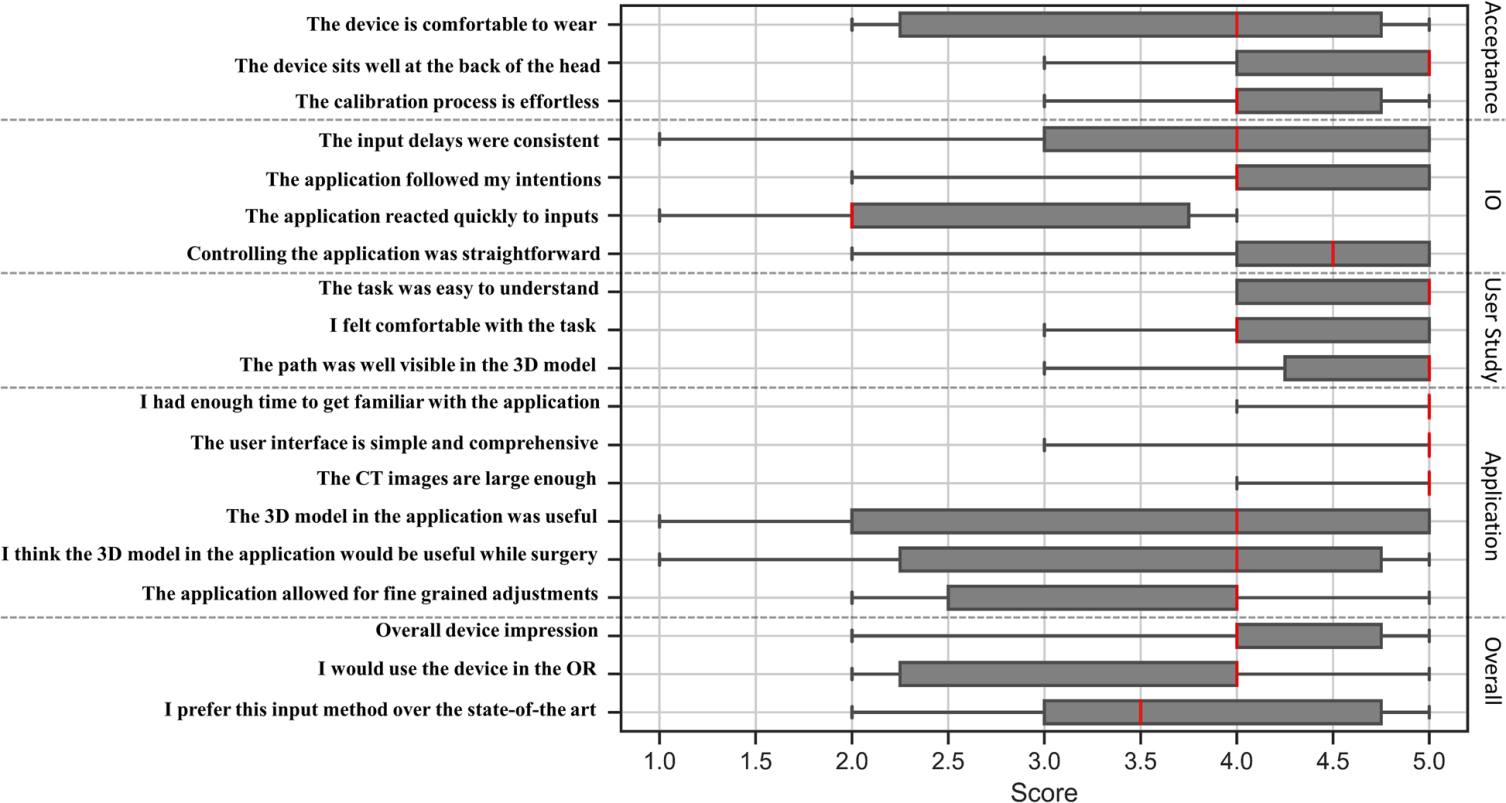
Participants' rating of the developed image control interface. Medians are displayed as red vertical lines. The individual box-plots depict IQR and whiskers show min and max

Through the qualitative evaluation of the participants, the average acceptability of the device was rated as 4.07 (SD: 0.96), the input and output as 3.65 (SD: 1.26), the user study as 4.47 (SD: 0.67), the application as 4.13 (SD:1.22) and the overall impressions of the interface as 3.77 (1.02). Details of the qualitative assessments are reported in Table 2 and Fig. 7.

**Table 2:** Participant's rating of the computer brain interface

Category	Question	Likert scale (Range: 1–5) Mean (SD)	
**Acceptance**	The device is comfortable to wear	3.6 (1.2)	4.1 (0.9)
	The device sits well at the back of the head	4.5 (0.67)	
	The calibration process is effortless	4.1 (0.7)	
**Input / Output**	The input delays were consistent	3.7 (1.3)	3.6 (1.2)
	The application followed my intentions	4.1 (0.9)	
	The application reacted quickly to inputs	2.6 (1.0)	
	Controlling the application was straightforward	4.2 (1.0)	
**User Study Task**	The task was easy to understand	4.6 (0.5)	4.5 (0.7)
	I felt comfortable with the task	4.2 (0.7)	
	The path was well visible in the 3D model	4.6 (0.7)	
**Application**	I had enough time to get familiar with the application	4.8 (0.4)	4.1 (1.2)
	The user interface is simple and comprehensive	4.7 (0.6)	
	The CT images are large enough	4.9 (0.3)	
	The 3D model in the application was useful	3.5 (1.5)	
	I think the 3D model in the application would be useful while surgery	3.4 (1.5)	
	The application allowed for fine grained adjustments	3.5 (1.0)	
**Overall** **Impressions**	Overall device impression	4.1 (0.8)	3.8 (1.0)
	I would use the device in the operation room	3.5 (1.1)	
	I prefer this input method over the state-of-the art	3.7 (1.0)

Through the Spearman's ranked correlation analysis, we identified a statistically significant correlation between the participants' calibration score and their acceptance rating (r_s_= 0.87; *p*=0.01) overall impression rating (r_s_= 0.66; *p* = 0.04)  while we found no statistically significant correlation between the participants' calibration score and their Input / Output rating, (r_s_= 0.61; *p*=0.06) user study rating (r_s_= 0.02; *p*=0.96) and application rating (r_s_= 0.59; *p*=0.07).

## Discussion

In this publication, we demonstrated the usability of a commercially available BCI device for the purpose of medical image control in intraoperative applications. For this, we designed a software interface that allowed for hands-free interaction with 3D medical imagery by following the commands of the users sensed by a head-mounted BCI device while not relying on specific physical gestures. We simulated an intraoperative image control task and environment and performed a user study to evaluate the feasibility of this interface in a simulated surgical setting. This early feasibility study showed that the existing limitations of currently available image control interfaces (e.g., sterility issues, line of sight problems and poor intuitiveness) can be facilitated through the use of the proposed interface.

As seen in Table 1, the participants could conduct the assigned image control tasks with a relatively low error rate. On average and for both of the tasks the participants' achieved an image control error of 15.5 mm (SD: 9.6). It should be noted that this error does not only stem from the developed interface but may also include the discrepancy in the participants' memorized trajectory and their executed trajectory as a confounding factor. Comparing this accuracy rate to the prior-art, to our knowledge most of the existing research on advanced image manipulation interfaces only report qualitative metrics or time of task completion (e.g., [9–11]) and lack quantitative analyses on spatial image control accuracy. However, compared to a study that implemented the closest counterpart to our 3D spatial accuracy metric [16] and despite the substantial differences in implementation of the metric and tasks, we found that our image control accuracy was better than the 3D target accuracy reported in that study (on average from 32.0 mm to 90.3 mm) despite the fact that our interface did not rely on any gesture recognition algorithms. We observed that the participant's error could be generally reduced if they spent more time in navigating to a specific target (Fig. 6). Furthermore, we did not observe a statistically significant difference between the two levels of task difficulty, which potentially demonstrate the insensitivity of the interface to the image control complexity (although a larger sample size is needed to derive conclusions to this end).

Based on the conducted user study, we showed that the participants perceived the developed interface as feasible in criteria such as acceptance and interface application design with respective average Likert scores of 4.1 and 4.1. We observed significant correlation between the participants' calibration scores and their acceptance and overall impression ratings, which demonstrates that the user experience can be improved if an adequate user-specific device calibration is accomplished.

While the user study provided valuable insight into the usability of the developed interface, our study cohort was rather small and therefore further investigation with a larger user study is planned for the future follow up studies. Furthermore, the image control tasks were performed in a simulated surgical setting that did not include some of the challenges that may arise inside a typical operating room (e.g., change in the lighting conditions, visual and auditory disruptions, etc.). To this end, our goal is to test the interface in real operating rooms in the future.

For a seamless integration of this interface in a surgical setting, several enhancements and modifications are required. The participants had a strong consensus on the slow response time of the device (average Likert score of 2.6), which can be noted as the most substantial limitation of the BCI device. Furthermore, although the utilized BCI sensor has a small form-factor, some of the participants expressed that they can envision ergonomic issues if this device is worn by the surgeon during long operations. Given that the utilized BCI is one of the earliest commercially available prototypes in the market, we hope that the proceeding generations of the product will have a quicker response time and smaller/lighter form-factor allowing for a more seamless image control experience.

## Conclusions

We believe that the intraoperative application of BCI for image manipulation is a viable option given that it can streamline the surgeons' commands when interacting with image display units. The developed interface can potentially reduce the surgical time by providing the surgeons with a direct communication channel with medical images. Similar BCI-based concepts can be investigated for other intraoperative tasks and more complex user interface algorithms such as physical controlling of surgical robots. 

## Supplementary Information

**Additional file 1. **Questionnaire.docx 

## Data Availability

The code for the developed software application can be downloaded from the link below: https://caspa.visualstudio.com/CARD%20public/_git/BrainInterfacePublic The datasets used and/or analyzed during the current study are available from the corresponding author on reasonable request.

## Abbreviations

2D: Two dimensional
3D: Three dimensional
BCI: Brain Computer Interface
CT: Computed Tomography
SD: Standard Deviation
MRI: Magnetic Resonance Imaging
PACS: Picture Archiving and Communication System
OR: Operating Room
WHO: World Health Organization
VEP: Visually Evoked Potential
SSVEP: Steady State Visually Evoked Potentials
SDK: Software Development Kit
API: Application Programming Interface

## Footnotes

1 https://www.slicer.org
