# Supplementary figures and images for: Introducing a brain-computer interface to facilitate intraoperative medical imaging control – a feasibility study
Source: BMC Musculoskelet Disord. 2022 Jul 22;23:701. doi: 10.1186/s12891-022-05384-9 (PMC9306028; doi:10.1186/s12891-022-05384-9)

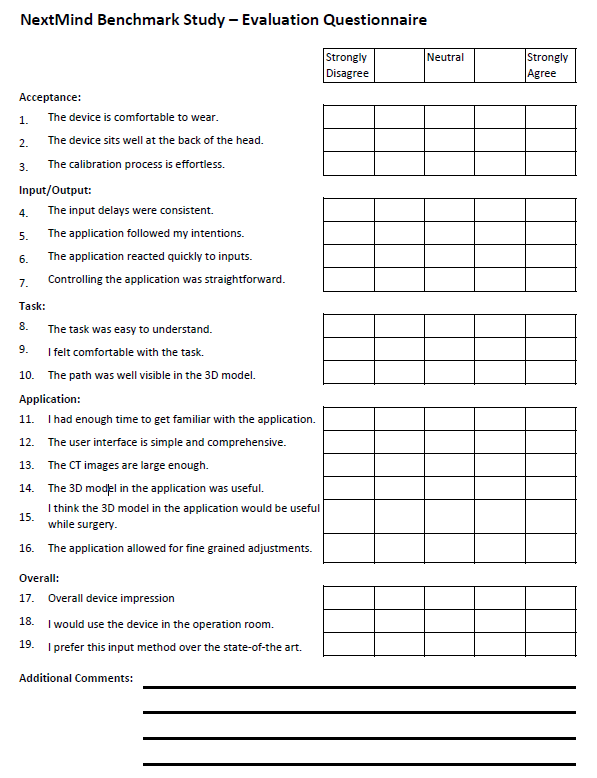

Supplement: Supplementary file 1 — Additional file 1. Questionnaire.docx [file 12891_2022_5384_MOESM1_ESM.docx]
